# Investigating ethnic differences in risk factors and severity of developing premature coronary artery disease: Predicting the effect of risk factors through decision tree analysis in a multicenter case-control study; Results from Iran Premature Coronary Artery Disease (IPAD study)

**DOI:** 10.34172/jcvtr.025.33190

**Published:** 2025-03-18

**Authors:** Seyed Ali Moezi Bady, Fatemeh Salmani, Ehsan Zarepur, Toba Kazemi, Neda Partovi, Nazanin Hanafi Bojd, Saeede Khosravi Bizhaem, Alireza Khosravi Farsani, Noushin Mohammadifard, Fereidoon Nouhi, Hassan Alikhasi, Masoumeh Sadeghi, Hamidreza Roohafza, Razieh Hassannejad, Katayoun Rabiei, Nahid Salehi, Kamal Solati, Masoud Lotfizadeh, Samad Ghaffari, Elmira Javanmardi, Arsalan Salari, Mostafa Dehghani, Mostafa Cheraghi, Habib Haybar, Reza Madadi, Nahid Azdaki, Nizal Sarrafzadegan

**Affiliations:** ^1^Cardiovascular Diseases Research Center, Birjand University of Medical Sciences, Birjand, Iran; ^2^Geriatric Health Research Center, Birjand University of Medical Sciences, Birjand, Iran; ^3^Interventional Cardiology Research Center, Cardiovascular Research Institute, Isfahan University of Medical Sciences, Isfahan, Iran; ^4^Department of Cardiology, Medicine School, Isfahan University of Medical Sciences, Isfahan, Iran; ^5^Hypertension Research Center, Cardiovascular Research Institute, Isfahan University of Medical Sciences, Isfahan, Iran; ^6^Pediatric Cardiovascular Research Center, Cardiovascular Research Institute, Isfahan University of Medical Sciences, Isfahan, Iran; ^7^The Iranian Network of Cardiovascular Research (INCVR), Iran; ^8^Rajaie Cardiovascular Medical and Research Center, Iran University of Medical Sciences, Tehran, Iran; ^9^Heart Failure Research Center, Cardiovascular Research Institute, Isfahan University of Medical Sciences, Isfahan, Iran; ^10^Cardiac Rehabilitation Research Center, Cardiovascular Research Institute, Isfahan University of Medical Sciences, Isfahan, Iran; ^11^Isfahan Cardiovascular Research Center, Cardiovascular Research Institute, Isfahan University of Medical Sciences, Isfahan, Iran; ^12^Cardiovascular Research Center, Health Institute, Kermanshah University of Medical Sciences, Kermanshah, Iran; ^13^Department of Psychiatry, Shahrekord University of Medical Sciences, Shahrekord, Iran; ^14^Social Determinants of Health Research Center, Shahrekord University of Medical Sciences, Shahrekord, Iran; ^15^Cardiovascular Research Center, Tabriz University of Medical Sciences, Tabriz, Iran; ^16^Department of Cardiovascular Medicine, Heart Center, Maragheh University of Medical Sciences, Amiralmomenin Hospital, Maragheh, Iran; ^17^Department of Cardiology, Healthy Heart Research Center, Heshmat Hospital, School of Medicine, Guilan University of Medical Sciences, Rasht, Iran; ^18^Department of Cardiovascular Research Center, Shahid Rahimi Hospital, Lorestan University of Medical Sciences, Khorramabad, Iran; ^19^Atherosclerosis Research Center, Ahvaz Jundishapur University of Medical Sciences, Ahvaz, Iran; ^20^Department of Cardiology, Zanjan University of Medical Sciences, Zanjan, Iran; ^21^Clinical Research Development Unit, Razi Hospital, Birjand University of Medical Sciences, Birjand, Iran; ^22^Faculty of Medicine, School of Population and Public Health, University of British Columbia, Vancouver, Canada

**Keywords:** Ethnic differences, Premature coronary artery disease, Decision tree, Multicenter, Case-control study

## Abstract

**Introduction:**

Premature coronary artery disease (PCAD) has an ascending trend especially in developing countries. This study have investigated the risk factors and severity of developing CAD across various Iranian ethnicities.

**Methods:**

This case-control study was done on 3015 Iranian patients undergoing coronary artery angiography, across highly populated Iranian ethnicities including Bakhtiari, Azari, Qashqai, Arab, Fars, Kurd, Gilak, and Lur. This study was performed over three years in 14 capitals of provinces in Iran headed by Isfahan Cardiovascular Research Center, by including men≤60 years old and women≤70 years undergoing coronary artery angiography. If they had coronary stenosis above 75% (more than 50% in the left main), they were categorized as Case group.The effects of conventional risk factors as well as psychosocial ones including age, gender, weight, Body mass index (BMI), economic status, cigarette smoking, drugs of abuse, stress, anxiety, diabetes, hypertension, etc. were determined in each ethnicity using decision tree statistical method. Also, via logistic regression method, the odds of incidence of CAD in each ethnicity were specified against the Fars ethnicity (the predominant ethnicity in Iran).

**Results:**

The most common risk factor among different ethnicities was age and male gender. Also, among the Iranian ethnicities, Kurd had the lowest chance while Gilak and Azari had the highest chance of developing PCAD as compared to the Fars ethnicity. Investigation of the behavioral and psychological dimensions indicated that stress was significantly higher among those without coronary artery involvement as compared to those with this involvement. The decision tree model could predict that among Gilakis, Fasting blood sugar (FBS) above 126 and in Lurs opium as well as diastolic blood pressure above 85, and in Kurds male gender would considerably increase the odds of developing CAD.

**Conclusion:**

The model obtained from the decision tree indicated that although variables of age, gender, cigarette, and opium are among the main risk factors for involvement of coronary arteries among young adult patients, in different ethnicities, the risk level of each of these risk factors in incidence of PCAD is different. This means among Kurds, age, among Gilakis diabetes, and among Lurs opium are more important.

## Introduction

 Coronary artery disease (CAD) is the most common type of cardiovascular disease and most important cause of mortality worldwide. Globally, this disease causes mortality with prevalence of 30%, which has been reported to be higher in Iran. More than 80% of CAD-related mortalities occur in low and middle-income countries.^1,2^ In Iran, as with most Western Asian counties, CAD is responsible for 46% of mortalities.^2^ In addition, the probability of premature morality between 30 and 70 years of age as a result of noncommunicable diseases, 48% of which being cardiovascular disease, is currently far higher in these regions as compared to western Europe or Northern America.^3^ Also, studies have shown that ethnicity can be effective in the incidence of this disease.^4^ Although patients with premature CAD have lower prevalence, the involvement in these patients has more grave consequence.^5^ Risk factors such as diabetes, hypertension, hyperlipidemia, and insufficient physical activity or sedentary lifestyle are effective in the prevalence of this disease. One of the possible risk factors alongside these risk factors is ethnicity; the different prevalence of disease across various counties confirms the probable trueness of this hypothesis. Iran is one of the very few countries with a very diverse range of ethnicities, including Bakhtiari, Qashqai, Azari, Arab, Fars, Kurd, Lur, and Gilak.^6^ Thus, this study can indicate the effect of ethnicity on CAD development. Considering the importance of ethnicity and its impact as well as its associated risk factors in CAD development, in Iran a study entitled “study on Iran premature coronary artery disease (IPAD) since 2020, whose results have been collected so far. It deals with examining ethnic differences in risk factor as well as severity of developing CAD among various Iranian ethnicities.^1^

 Different methods are used to determine the risk factors of different diseases, one of them is the decision tree method,in this study decision tree method used to determine CAD risk factors, which is among the most famous and oldest methods of classification model development. In these algorithms, the data are usually presented as an algorithm whose results are interpretable. A key advantage of decision trees is that they can be used in graphical forms.^7^ In different studies, decision tree has been used for CAD prediction. For example, Joloudari et al^8^ considered this method suitable for determining CAD risk factors.

 A multicenter case-control study works by involving a larger and more diverse group of participants to reduce bias and increase the validity of the results. Since CAD is a common and complex disease that is influenced by a wide range of factors, including genetic, environmental, and lifestyle factors, conducting a multicenter case-control study can help to increase the sample size and diversity of the study population, which can improve the statistical power of the study and increase the likelihood of identifying significant risk factors. Additionally, involving multiple centers can help to ensure that the findings are applicable across different regions and populations, which can be particularly important when studying a disease as complex and multifactorial as CAD.

 The aim of this study was to investigate important predictors of CAD in different Iranian ethnic groups, as these groups live in different parts of the country. Therefore, we explored the effect of ethnic risk factors though decision tree algorithm in several capitals of provinces in Iran using a multicentric case-control study.

## Materials and Methods

###  Design and aims

 IPAD is a multicenter case-control study performed on patients with various Iranian ethnicities in more than ten cities in Iran from May 2019 to March 2021 with centrality or supervision and leadership of Isfahan cardiovascular research center as well as cooperation of 14 center of the country including,Isfahan (1), Tabriz (2), Maragheh (3), Kermanshah (4), Khorramabad (5), Ahvaz (6), Shahrekord (7), Zanjan (8), Zahedan (9), Birjand (10), Yazd (11), Shiraz (12), Rasht (13), Birjand (14). The sample allocation was based on the proportion of each ethnicity in Iran, where 58% were Persian, 13% were Kurdish, 8% were Gilaki, 7% were Bakhtiari, Qashqai, and Lur, each comprising 4% of the total population under study, and 3% were Azerbaijani and Arab, respectively.

 These patients include men and women who had undergone coronary artery angiography (IPAD methodology paper)1. After acquiring ethics code with the No. IR.MUI.REC.1396.2.055, it was performed on patients undergoing coronary artery angiography including all women ≤ 70 years and men ≤ 60 years. They were assigned into case (with positive result in coronary artery angiography, minimum stenosis of one artery above 75% or left main more than 50%) and control (with normal result of coronary artery angiography). Previous history of documented coronary artery disease such as coronary artery bypass surgery, balloon angioplasty, or percutaneous coronary intervention (PCI) was considered as exclusion criterion.After acquiring written informed consent form, variables of age, gender, ethnicity, religion, levels of education, income, occupational status, history of cigarette smoking, alcohol consumption, addiction, along with personal and familial history of CVD, drug consumption, behavioral habits and extent of physical activity, were collected in fata collection forms by trained inquirers.

 Coping with stress scale and depression and anxiety (HADS) questionnaire were also completed for the patients.

 It consisted of the 23 items divided into five part: Positive reinterpretation and growth, Problem engagement, Acceptance, seeking support and Avoidance. The reliability of the questionnaire was determined using Cronbach’s alpha coefficient (a = 0.84). Each item was scored on a 3-point scale (never = 0, sometimes = 1, and often = 2). For each scales, separate scores were reported.1

 Heart rate was measured during one minute, while systolic and diastolic blood pressure was measure twice with 15-min interval, in a sitting position under standard conditions. Anthropometric measurements including height, weight, wrist circumference, neck circumference, thigh circumference, waist circumference, and hip circumference were also measured (all in cm).

 The serum sample was collected for FBS, LDL, HDL, and Chol tests. They were transferred from all study sites to the central laboratory of Isfahan CVD research center once every few months by a contracted transportation company. They were frozen and kept at -70˚C for subsequent genetic* tests, biobank formation, and epigenic studies.

 The data collectors in each site received the necessary training in some sessions, where the supervision committee supervised the method of implementation of the protocol through numerous trips. All information taken from patients was transferred to the data management center of Isfahan CVD research center.

###  Inclusion and exclusion criteria

 The inclusion criteria were female patient equal to or ≤ 70 years, or male patient equal to or younger than 60 years, identical father and mother ethnicity, minimum stenosis of one artery above 75% (or left main more than 50%), who would be assigned to the case group, while the normal individuals were assigned to the control group. History of PCI and coronary artery bypass graft (CABG) were among the exclusion criteria.

###  Statistical Analysis 

 In this study, information was recorded based on identical questionnaires by trained interviewers. Then, all questionnaires and serum samples were sent to the Isfahan Cardiovascular Research Center. All questionnaires were entered into the software using the same protocol. Data exploration, error correction, and identification of incorrect information were performed at the center. Finally, all data from all ethnic groups were analyzed as one dataset using IBM SPSS Modeler 18.0 quantitative variables were described using mean and standard deviation, and qualitative variables were described using count and percentage. Logistic regression model with Fars ethnicity as the reference was used to compare the odds of CAD occurrence in different ethnic groups. Independent t-test was used for comparing the mean of quantitative variables between patients with and without CAD in each ethnicity. Additionally, the chi-square test was used to examine the relationship between qualitative variables and CAD occurrence.

 Decision tree theory was used to simultaneously investigate the effect of study variables (demographic, clinical, etc.). Decision tree theory enabled the detection of complex interactions and non-linear relationships between the study variables that may have been missed by traditional logistic regression methods. A decision tree model was constructed, with demographic, clinical, and other study variables as predictors and CAD occurrence as the outcome variable. Decision Tree analysis was employed by using the Chi-Square Automatic Interaction Detection (CHAID). CHAID is a decision tree technique that allows for the creation of multiple child nodes at each split, rather than being limited to binary splits. It works by identifying interactions among the input features and using chi-square tests to find the best multi-way split for each node. The splits are selected to maximize the statistical significance of the relationship between the input features and the target variable.

 In essence, CHAID trees are capable of detecting feature interactions and performing multi-way splits based on statistical associations. The chain approach was used to model label dependencies by sequentially fitting multiple binary classifiers, where the prediction of one label influences the prediction of the subsequent labels. This strategy helps improve predictive accuracy in complex, multi-label classification tasks.

 To evaluate the performance of the model, the accuracy was used as the primary performance metric. Accuracy, defined as the proportion of correctly classified instances over the total number of instances. A significance level of *P* < 0.05 was used to determine statistical significance.

## Results

 In this study, 3015 individuals participated. The mean age of the participants was 53.72 ± 7.69 years, with 1637 individuals being male (54.3%). Most participants (89.4%) were married and with primary education (30.2%). Among them, 1819 (60.3%) had CAD.

**Figure 1:**
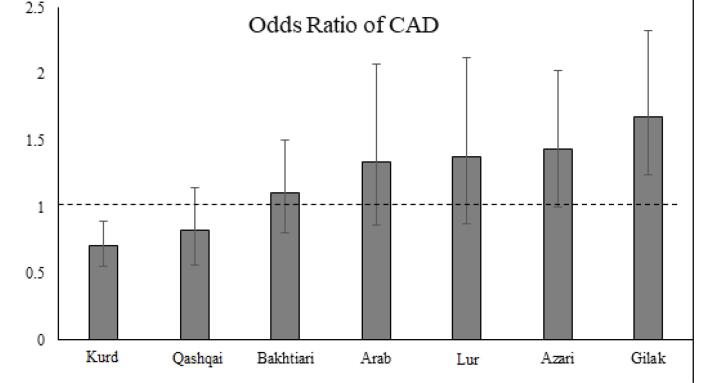
Chances of getting CAD in different ethnic groups compared to Fars people

 Initially, the Odds ratio of CAD was calculated for different ethnicities compared to the Fars ethnicity using logistic regression. As a significant percentage of the population belongs to the Fars ethnicity, it was considered as the reference. Based on the results, the ethnicity can have a significant effect on CAD occurrence (*P* < 0.05). Figure 1 shows the odds ratio and 95% confidence interval of CAD in each ethnicity compared to the Fars ethnicity. The odds ratio of getting of PCAD was significantly higher among Gilak compared to Fars ethnicity (OR = 1.68, CI95%: 1.24- 2.27). Also, Azari ethnicity had higher odds (OR = 1.44. CI95% = 0.94-2.19) compared to the Fars ethnicity. However, Kurd (OR = 0.71; CI95% = 0.56-0.89) and Qashqai (OR = 0.82, CI95%: 0.57-1.18) had significantly lower odds of developing PCAD compared to Fars.

 Since the chance of CAD occurrence may vary among different ethnicities in the present study, we have examined the role of each demographic and clinical variable on CAD according to each ethnicity. on the results of independent t-tests and chi-square tests presented in Supplementary file, Table S1-S8 in addition odds ratio and related confidence intervals were reported.

 The age variable had a significant difference in all ethnic groups (Fars: OR = 1.04, *P* < 0.001, Gilak: OR = 1.06, *P* < 0.001, Kurd: OR = 1.07, *P* < 0.001, Arab: OR = 1.08, *P* < 0.001, Lur: OR = 1.06, *P* = 0.004, Bakhtiari: OR = 1.06, *P* = 0.002) except for the Qashqai and Azari ethnicity among patients with and without CAD, with older individuals being more likely to be in the CAD group.

 Additionally, being male was more common in all ethnic groups (Fars: OR = 4.08,*P* < 0.001, Azari: OR = 3.16, *P* = 0.008, Gilak: OR = 1.85, *P* = 0.039, Kurd: OR = 2.48, *P* < 0.001, Qashqai: OR = 4.22, *P* < 0.001, Bakhtiari: OR = 6, *P* < 0.001) except for Arab and Lur among patients with CAD.

 The use of opium was more prevalent among the Bakhtiari (OR = 2.65, *P* = 0.005), Fars (OR = 2.50, *P* < 0.001), Kurd (OR = 1.88, *P* = 0.018), and Lur (OR = 4.43, *P* = 0.04) ethnicities among CAD patients. However, smoking was specifically identified as a risk factor among the Arab ethnicity (OR = 4.78, *P* = 0.012).

 Moreover, high LDL in the Bakhtiari (OR = 0.3, *P* = 0.003), Fars(OR = 0.48, *P* < 0.001), Kurd (OR = 0.47, *P* = 0.022), and Lur(OR = 0.18, *P* = 0.009) ethnicities could also be a predictor for CAD status.

 In addition, BMI (OR = 0.93, *P* = 0.023), abnormal waist circumference (OR = 0.47, *P* = 0.014), high diastolic blood pressure (OR = 1.04, *P* = 0.004) can be predictors for CAD development among Bakhtiari ethnicity.

 High systolic hypertension (OR = 1.01, *P* = 0.001), history of diabetes (OR = 1.57, *P* < 0.001), high FBS (OR = 2.22, *P* < 0.001) were among disease risk factors in the Fars ethnicity. These risk factors were also present for other ethnicities individually.

 Since demographic and clinical variables can have different effects on disease occurrence when considered together, here we have used decision tree analysis to simultaneously examine the role of study variables in each ethnicity. Decision trees can handle interactions between variables more easily than logistic regression and provide a graphical representation of the decision-making process, which can be easier to interpret.

 Decision tree analysis was performed for each ethnicity, and the accuracy of the analysis for each ethnicity is presented in (Table 1). The analysis accuracy ranged from 65 to 85%, and was above 70% on average for all ethnicities, showing reliability of this tree model. All decision trees showed sensitivity values above 80%. Additionally, the positive predictive value of the models, except for the Kurdish ethnicity, was above 70% in all models. The specificity of the presented models was moderate, with the best specificity reported in the Persian group. The negative predictive value of the models, except for the Lur ethnicity, was above 50%.

**Table 1:** Accuracy of decision tree analysis for different ethnicities

**Ethnicity**	**Sensitivity**	**Specificity **	**PPV**	**NPV**	**Accuracy**
Fars	0.83	0.61	0.77	0.70	72.67
Azari	0.88	0.40	0.76	0.60	69.16
Gilak	0.88	0.41	0.80	0.57	84.23
Kurd	0.82	0.55	0.67	0.74	77.84
Arab	0.85	0.55	0.80	0.63	76.29
Lur	0.88	0.43	0.86	0.50	79.1
Qashqai	0.90	0.54	0.71	0.81	65.65
Bakhtiari	0.89	0.52	0.76	0.73	68.09

PPV: Positive Predictive value, NPV: Negative Predictive value

 Based on the results of decision tree, in the Fars ethnicity, men have been more involved with PCAD than women, and following gender, age is the most important determining risk factor; the odds of disease development have been higher in men above 54 years. However, in men between 43 and 54 years, HDL < 40 increases the odds of disease development.

 In the Kurd ethnicity, the most important determinant risk factor was age, where those between 55 and 59 years had the highest odds of disease development. In those younger than 49 years, those with sleep quality lower than 7 had a higher risk level compared to their peers. For those above 59 years, HTN is considered a risk factor. In this ethnicity, male gender would increase the odds of disease development.

 In Gilak ethnicity, FBS > 126 is a risk factor for CAD development. In the Bakhtiari ethnicity, men above 47 years are at risk of disease, and in Qashqai ethnicity, men with HTN history had higher odds of disease development.

 In Lur ethnicity, addicts younger than 53 years with diastolic blood pressure above 85 have higher odds of CAD. The referring patients with systolic blood pressure above 115 and diastolic blood pressure lower than 82.5 have higher odds of disease development.

 Azari men and the Arabs with social support lower than 8, as well as the cigarette smokers having social support score above 11 had high risk of disease development.

**Figure 2:**
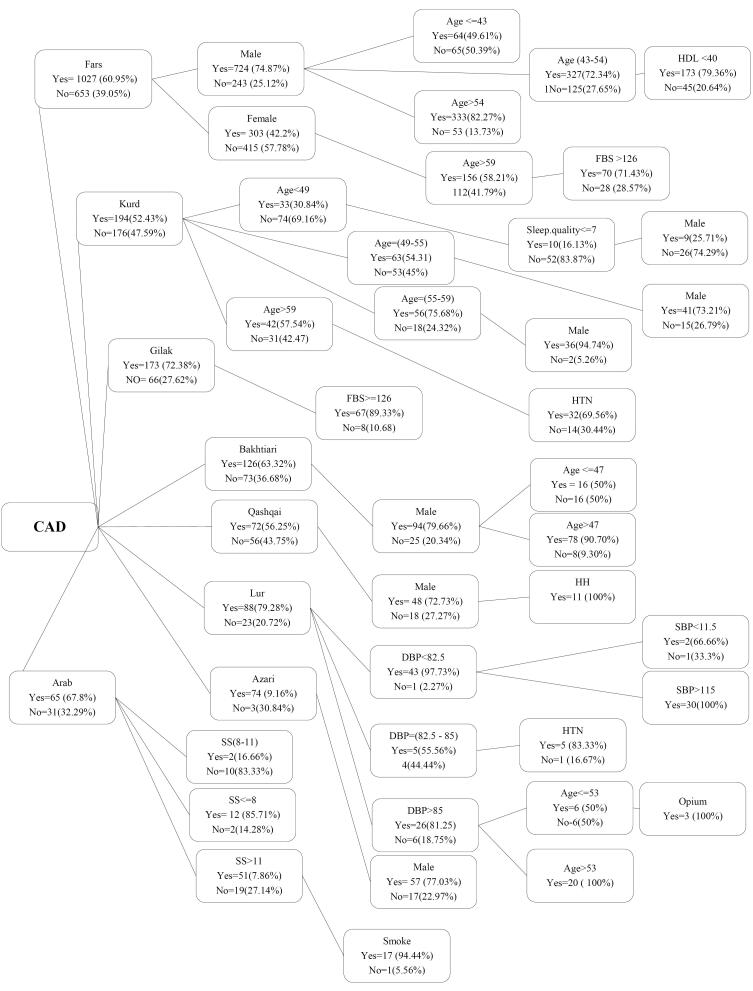
Display of Decision Tree with three initial variables by ethnicity

 Also, based on analyzing the accuracy of decision tree method, its analysis accuracy ranged from 65 to 85%, and was above 70% on average for all ethnicities, showing reliability of this tree model (Figure 2).

## Discussion

 PCAD incurs various clinical consequences and socioeconomic costs. Studies on the effect of race and ethnicity have been performed in developed countries, though mostly on white and black races, or various Latino, Asian, and Spanish nationalities. Past studies have mentioned Asian race as the one with a higher risk of premature CAD.^5,9^ Thus, it can be stated that our study can further clarify the status of PCAD in Asia. Iran, considering its wide geographical scope, is composed of various ethnicities including Azari, Kurd, Lur, Gilaki, Arab, Bakhtiari, Azari, and Fars. Some of these ethnicities such as Kurd, Azari, and Arab are shared between Iran and neighboring countries including Iraq, Azerbaijan, etc. Thus, investigation of ethnic risk factors can be generalized to neighboring countries. The study by Ameen Mosa Mohammad et al in Kurdistan, Iraq, which was on the prevalence of PCAD risk factors, based on logistic regression model, they stated that male gender, hyperlipidemia, cigarette, and hypertension had a significant association with the severity and involvement of coronary arteries in Iraqi Kurd ethnicity, which is in line with our study regarding the variables of gender, cigarette, and dyslipidemia. The notable point was significance of opium along with psychological factors such as anxiety and depression in our study, which has not been investigated in this ethnicity so far. Thus, these factors in addition to the three factors of gender, cigarette, and dyslipidemia can be considered as risk factors in the Kurd ethnicity. The factor of hypertension in the study by Ameen et al was a risk factor in Iraqi Kurd patients, which was not found to be significant in our study, possibly due to the type of culture, lifestyle, and diet of these two groups.^10^

 With regards to Arab ethnicity, in the study by Reda et al^11^ cigarette, female gender, diabetes, and obesity were found as risk factors of Arab race in incidence of premature CAD, which was in line with our study regarding gender and cigarette variables.

 In another study in 2024 with the title of identifying the risk factors and mortality rates due to premature coronary artery disease in the young Saudi population. Smoking, diabetes, hypertension, family history of CAD, dyslipidemia, and overweight/obesity are significantly and positively associated with premature coronary artery disease.

 About 5–10% of all acute myocardial infarction (AMI) cases occur in patients younger than 45 years. These percentages are higher in Middle Eastern countries (11%) than Western Europe (2.7%), North America (4%) and Africa (9%). As a primary risk factor, smoking plays an important role in premature coronary atherosclerosis and in accelerating atherosclerosis by increasing the oxidation of low-density lipoprotein (LDL) and injury coronary endothelial vasodilation. In this context, many studies have shown that smoking is significantly associated with an increased risk of PCAD compared to healthy subjects. The results of our study showed that cigarette smoking is an important factor in relation to early coronary artery involvement in all population except Lur.^12,13^

 Considering Azari ethnicity,^14^ they had the highest odds ratio of developing premature CAD among the European societies, reflecting the importance of Turk ethnicity in incidence of PCAD. The study by Conkbayir^15^ described cigarette, hypertension, and diabetes as the risk factors of these patients, while high cholesterol, unlike common studies, was not a risk factor for CAD development. The notable point is the insignificance of high cholesterol in Iranian Azari ethnicity as a risk factor and the concordance of these results with the findings for Azari patients in turkey.

 In the study by Abbasi et al on 20165 patients from Fars, Kurd, Gilaki, Mazani, Lur, and Kurd ethnicities, the number of risk factors was considered from 0 to 4, and more than 4; it has been stated that in all ethnicities of Fars, Azari, Gilaki, Mazani, Lur, and Kurd, male gender was found as a risk factor for developing CAD; in our study, out of eight groups, in six of them, male gender was found as a risk factor. Except for Arab and Lur ethnicities, this difference can be attributed to aggregation of these groups in the study by Abbasi as ‘other groups’, and combining the results of ethnicities in this form. In the study by Abbasi, Gilakis smoked less cigarette than others, which was in line with our study, since cigarette smoking has not been stated as a significant variable for Gilaki ethnicity. On the other hand, in other ethnicities, cigarette and opium were among the significant risk factors for CAD development. One of the significant risk factors for Gilakis in our study was hypertension; in the study by Abbasi although it was higher in Mazani people, Gilakis had more cases of hypertension compared to Fars. Another risk factor in our study was having diabetes, which was significant among Gilakis, just as in Abbasi study in which the highest rate of diabetes was related to Gilaki ethnicity. In our study, the lowest odds of CAD development were found among Lurs; in spite of higher prevalence of opium use and high cholesterol than other Iranian ethnicities, their lifestyle, physical activity, and type of diet have differentiated them from other ethnicities.^6^

 In the study, examining diabetes across Fars, Arab, Kurd, Lur, and Bakhtiari ethnicities, they introduced Fars ethnicity as the one with the highest cases of diabetes, while the lowest was found for Bakhtiari, which is in line with our study.^16,17^

 In the study by Allahyari et al exploring depression across various Iranian ethnicities, Kurd ethnicity following Baluch had the highest rate of depression compared to Lur, Azari, Arab, and Turkman, which concurs with our study.^18^ In our study, in the Kurd ethnicity, depression was found as one of the significant variables in PCAD development. This parameter may be regarded as a reason for higher odds of CAD development among Kurds. Thus, special attention should be paid to these regions regarding methods of preventing and treating depression as well as resolving its roots.

 Although stress, anxiety, and depression have been known as risk factors for CAD, in our study due to earlier and more frequent referral of these patients for undergoing angiography, PCAD prevalence was lower among them compared to those without psychological issues. This has been in line with the study by Wheeler et al, examining chest pain in patients with CAD and without it. He stated that incidence of depression was 63% in those without CAD, while it was 53% in those with CAD. As a justification, it may be stated that these patients referred following the severity of stress and anxiety that induced palpitations and chest pain. Meanwhile, based on previous studies by Khayaga, who suggested that depression and stress would increase the chance of developing heart attack, in patients without CAD who have anxiety, stress, and depression, root cause analysis is essential in order to prevent CVD.^19^

 Another risk factor related to CAD was BMI; in our study Gilakis showed it as a risk factor for developing CAD. This study was in line with Farzadfar et al who found Mazandaran province as the one with the highest percentage of obesity.^20^

 The limitations included being multicenter, the variety of inquirers, as well as the manner and extent of patient cooperation in responding to the numerous questions in the diet and psychosocial questionnaires, as well as time-consuming nature of interviews plus data collection.

 If the severity of coronary artery involvement and their complexity are examined with syntax score in all provinces and ethnicities, the effect of ethnicities on the extent and severity of atherosclerosis could also be investigated.

## Conclusion

 The model obtained from the decision tree indicated that although variables of age, gender, cigarette, and opium are among the main risk factors for involvement of coronary arteries among young adult patients, in different ethnicities, the risk level of each of these risk factors in incidence of PCAD is different. This means among Kurds, age, among Gilakis diabetes, and among Lurs opium are more important. According to the results of this study, health officials are suggested to plan in relation to the prevention of premature coronary artery disease according to the type of race.

## Competing Interests

 The authors declare no conflict of interest.

## Ethical Approval

 The patients/participants provided their written informed consent to participate in this study. The protocol of study was approved by ethics code with the No. IR.MUI.REC.1396.2.055(Research Ethics Committees of Isfahan University of Medical Sciences and Health Services, Iran)

## Supplementary Files


Supplementary file 1 contains Table S1-S8.
