# Supplementary material for: Investigating ethnic differences in risk factors and severity of developing premature coronary artery disease: Predicting the effect of risk factors through decision tree analysis in a multicenter case-control study; Results from Iran Premature Coronary Artery Disease (IPAD study)
Source: J Cardiovasc Thorac Res. 2025 Mar 18;17(1):49–57. doi: 10.34172/jcvtr.025.33190 (PMC12068799; doi:10.34172/jcvtr.025.33190)

J Cardiovasc Thorac Res. 2025;17(1):49-S1
doi: 10.34172/jcvtr.025.33190
http://jcvtr.tbzmed.ac.ir

Table S1: Comparison of demographic variables and clinical characteristics between individuals with and without CAD in the Bakhtiari ethnic group

Variables		With CAD	Without CAD	P-Value	Odds ratio and 95% confidence interval		
					OR	25%	97.5%
Age		54.23±6.7	50.86±8.48	0.002	1.06	1.02	1.11
BMI		27.3±4.2	28.9±5.4	0.023	0.93	0.87	0.99
WC		65 (52)	51 (69.9)	0.014	0.47	0.25	0.86
SBP		123.9±20.1	120±17	0.173	1.01	1.00	1.03
DBP		80.2±11.2	75.4±11.5	0.004	1.04	1.01	1.07
Sex (female) n(%)		32 (25.4)	49 (67.1)	<0.001	6	3.19	11.28
Sex (male) n(%)		94 (74.6)	24 (32.9)				
Economic		5.7±2.03	4.9±2.2	0.02	1.18	1.02	1.36
Smoke n(%)		66(52.4)	17(23.3)	<0.001	3.62	1.90	6.91
Opium n(%)		46(36.5)	13(17.8)	0.005	2.65	1.32	5.35
Stress	SSS	9.2±4.2	8.6±4.5	0.388	1.03	0.96	1.10
	PE	8.2±3.4	8.9±3.1	0.148	0.93	0.85	1.02
	PG	5.8±1.2	5.7±2.1	0.677	1.03	0.89	1.20
	Acceptance	2.9±1.2	2.9±1.3	0.927	0.99	0.79	1.24
	Avoidance	3.5±2.2	3.4±2.4	0.679	1.03	0.90	1.17
Anxiety.		9.6±5.9	11.2±6	0.061	0.95	0.91	1.00
Depression		8.5±4.6	9.4±4.5	0.173	0.96	0.90	1.02
Sex activity		6.7±1.8	6±2.2	0.024	1.18	1.02	1.37
DM n(%)		33 (28.2)	21 (30.9)	0.699	0.88	0.46	1.69
High FBS n(%)		21(18.6)	10(15.2)	0.558	1.28	0.56	2.91
High LDL n(%)		11 (8.9)	18 (24.7)	0.003	0.3	0.13	0.67
High Chol n(%)		6 (4.8)	4 (5.5)	0.833	0.87	0.24	3.19
High TG n(%)		32 (25.8)	18 (25.4)	0.944	1.02	0.52	2.00
Low HDL n(%)		90 (72.6)	54(74)	0.832	0.93	0.48	1.79
DLP n(%)		103 (83.1)	64 (87.7)	0.385	0.69	0.30	1.60
HTN n(%)		51(40.5)	27(37)	0.627	1.16	0.64	2.10
History DLP n(%)		43(34.1)	27(37)	0.684	0.88	0.48	1.61
History DM n(%)		30(24)	17(23.3)	0.91	1.04	0.53	2.05
History HTN n(%)		37(29.4)	22(30.1)	0.909	0.96	0.51	1.81

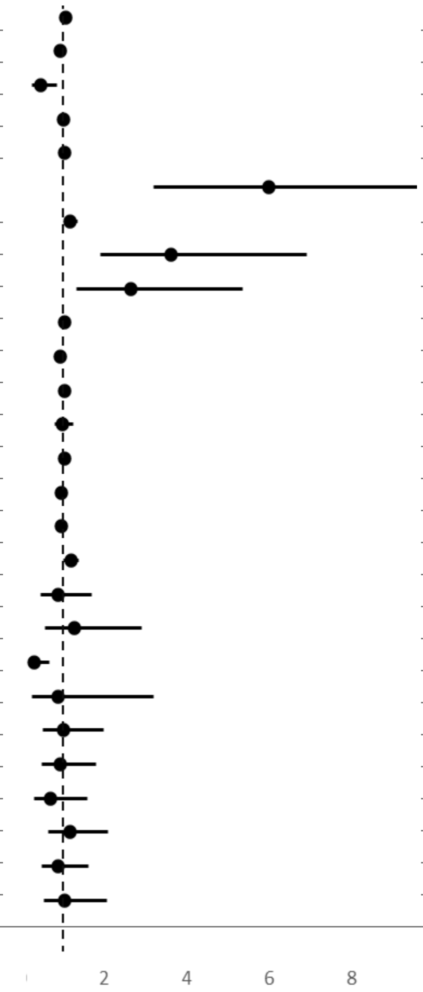

Table S2: Comparison of demographic variables and clinical characteristics between individuals with and without CAD in the Azari ethnic group

Variables		With CAD	Without CAD	P-Value	Odds ratio and 95% confidence interval OR	25%	97.5%
Age		54.15±8.87	52±8.73	0.11	1.03	0.98	1.08
BMI		28.3±3.2	29.2±5.1	0.303	0.93	0.84	1.04
WC		53 (77.9)	22 (71)	0.453	1.45	0.55	3.79
SBP		122.4±15.01	121.8±13.7	0.855	1	0.97	1.03
DBP		77.8±10.9	77.6±8.8	0.924	1	0.96	1.04
Sex (female) n(%)		17 (23)	16 (48.5)	0.008	3.16	1.32	7.54
Sex (male) n(%)		57 (77)	17 (51.5)				
Economic		6.6±2.9	6.4±2.05	0.566	1.04	0.89	1.21
Smoke n(%)		35(47.3)	11(33.3)	0.178	1.79	0.76	4.22
Opium n(%)		5 (6.8)	4(12.1)	0.356	0.53	0.13	2.1
Stress	SSS	8.5±4.4	8.1±4.5	0.646	1.02	0.93	1.12
	PE	8.2±2.8	8.8±2.4	0.245	0.91	0.77	1.07
	PG	5.5±2.1	5.3±2.2	0.683	1.04	0.86	1.26
	Acceptance	2.9±1.2	3.2±1.04	0.268	0.8	0.55	1.18
	Avoidance	2.2±1.8	1.3±1.38	0.019	1.38	1.05	1.82
Anxiety.		7.5±4.4	9.5±4.5	0.042	0.91	0.84	1
Depression		5.9±3.5	7.5±4.4	0.055	0.9	0.81	1
Sex activity		5.5±1.9	6.3±1.7	0.054	0.79	0.61	1.01
DMn(%)		27 (71.1)	8 (80)	0.571	0.61	0.11	3.36
High FBSn(%)		9 (45)	0(0)	0.217	-	-	-
High LDL n(%)		8 (16)	2 (12.5)	0.734	1.33	0.25	7.04
High Chol n(%)		2 (3.8)	1 (6.3)	0.682	0.6	0.05	7.09
High TG n(%)		6 (11.5)	3 (18.8)	0.475	0.57	0.12	2.58
Low HDL n(%)		30 (60)	9 (56.3)	0.791	1.17	0.37	3.64
DLP n(%)		49 (81.7)	19 (79.2)	0.792	1.17	0.36	3.82
HTN n(%)		37(50)	18(54.5)	0.664	0.83	0.37	1.9
History DLP n(%)		36(48.6)	14(43.8)	0.643	1.21	0.529	2.805
History DM n(%)		26 (35.1)	8(24.2)	0.264	1.69	0.67	4.28
History HTN n(%)		29(39.2)	16(48.5)	0.368	0.68	0.3	1.57

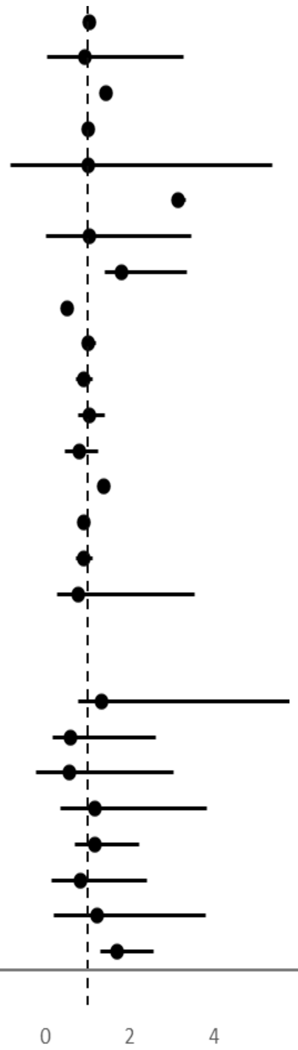

TableS3: Comparison of demographic variables and clinical characteristics between individuals with and without CAD in the Qashqai ethnic group

Variables		With CAD	Without CAD	P-Value	Odds ratio and 95% confidence interval		
					OR	25%	97.5%
Age		54.85±7.05	52.79±7.25	0.09	1.04	0.99	1.10
BMI		28±4.3	28.2±4.8	0.781	0.99	0.92	1.07
WC		41 (57.7)	39 (69.6)	0.168	0.60	0.28	1.25
SBP		125.3±2.3	125.1±16.7	0.951	1.00	0.98	1.02
DBP		80.1±14.4	77.6±11.5	0.285	1.01	0.99	1.04
Sex (female) n(%)		24 (33.3)	38 (67.9)	<0.001	4.22	2.01	8.89
Sex (male) n(%)		48 (66.7)	18 (32.1)				
Economic		6.02±1.9	6.3±2.2	0.395	0.93	0.78	1.10
Smoke n(%)		41(56.9)	18(32.1)	0.005	2.79	1.35	5.79
Opium n(%)		24(33.3)	11(19.6)	0.085	2.05	0.90	4.65
Stress	SSS	8.4±4.3	9.7±4.2	0.14	0.93	0.86	1.01
	PE	8.1±3.7	7.4±4.07	0.319	1.05	0.96	1.15
	PG	5.8±2.05	5.9±2.07	0.761	0.97	0.82	1.16
	Acceptance	2.7±1.2	2.7±1.3	0.985	1.00	0.77	1.31
	Avoidance	3.7±1.9	3.2±2.2	0.605	1.05	0.88	1.24
Anxiety.		8.7±5.6	11.3±5.3	0.01	0.92	0.86	0.98
Depression		8.1±4.2	9.1±4.7	0.185	0.95	0.88	1.03
Sex activity		6.2±2.05	5.6±2.6	0.146	1.11	0.95	1.30
DM n(%)		17 (25)	10 (20)	0.523	1.33	0.55	3.23
High FBS n(%)		13(19.1)	5(10.2)	0.187	2.08	0.69	6.28
High LDL n(%)		6 (8.5)	8 (14.3)	0.297	0.55	0.18	1.70
High Chol n(%)		2 (2.8)	5 (8.9)	0.129	0.29	0.05	1.56
High TG n(%)		14 (19.4)	10 (18.2)	0.857	1.09	0.44	2.67
Low HDL n(%)		50 (69.4)	35 (62.5)	0.409	1.36	0.65	2.85
DLP n(%)		57 (80.3)	41 (73.2)	0.346	1.49	0.65	3.42
HTN n(%)		37(51.4)	23(41.1)	0.246	1.52	0.75	3.07
History DLP n(%)		25(34.7)	15(26.8)	0.237	0.69	0.32	1.48
History DM n(%)		11(15.3)	7(12.5)	0.654	1.26	0.46	3.50
History HTN n(%)		26(36.1)	15(26.8)	0.262	1.54	0.72	3.31

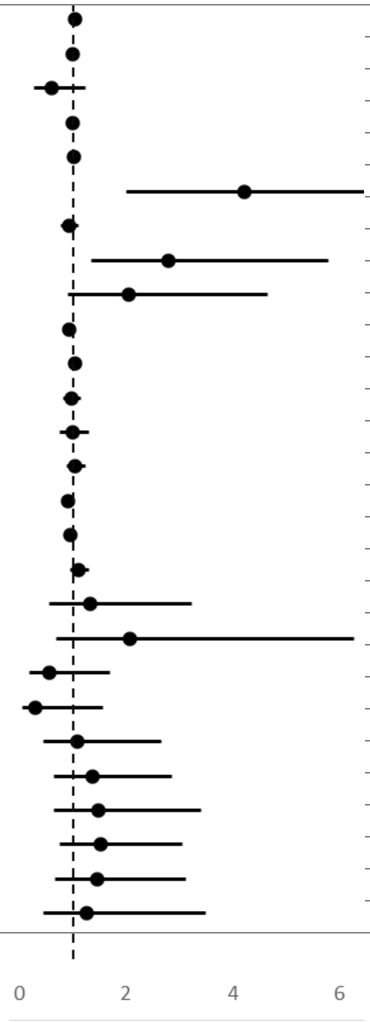

TableS4: Comparison of demographic variables and clinical characteristics between individuals with and without CAD in the Arab ethnic group

Variables		With CAD	Without CAD	P-Value	OR	25%	97.5%
					Odds ratio and 95% confidence interval		
Age		53.98±7.26	49.16±9.17	<0.001	1.08	1.02	1.14
BMI		27.8±4.7	28.5±4.9	0.521	0.97	0.89	1.06
WC		44 (68.8)	23 (74.2)	0.585	0.77	0.29	2.00
SBP		129.4±17.3	124.2±13.4	0.458	1.02	0.97	1.08
DBP		79.2±10.2	79.5±12.5	0.958	1.00	0.92	1.08
Sex (female) n(%)		34 (52.3)	22 (71)	0.083	2.23	0.89	5.57
Sex (male) n(%)		31 (47.7)	9 (29)				
Economic		7.3±2.1	6.9±2.4	0.515	1.07	0.87	1.32
Smoke n(%)		22(33.8)	3(9.7)	0.012	4.78	1.31	17.47
Opium n(%)		10(15.4)	1(3.2)	0.08	5.45	0.67	44.69
Stress	SSS	10.6±4.3	11.2±2.5	0.418	0.95	0.82	1.10
	PE	9.2±2.7	8.9±2.5	0.738	1.03	0.86	1.25
	PG	6.6±1.6	6.9±1.4	0.519	0.89	0.64	1.25
	Acceptance	3.2±0.98	3.2±1.2	0.943	1.02	0.63	1.64
	Avoidance	3.4±1.9	3.4±1.9	0.89	0.98	0.75	1.28
Anxiety		11.4±5	11±5.07	0.74	1.02	0.93	1.11
Depression		8.2±3.9	8.4±3.2	0.866	0.99	0.88	1.11
Sex activity		6.8±2	6.6±1.1	0.731	1.11	0.64	1.94
DM n(%)		26 (89.7)	6 (66.7)	0.098	4.33	0.70	27.01
High FBS n(%)		3(50)	0(0)	0.134			
High LDL n(%)		0 (0)	1 (33.3)	0.087			
High Chol n(%)		0 (0)	1 (33.3)	0.087			
High TG n(%)		0 (0)	1 (33.3)	0.107			
Low HDL n(%)		2 (25)	2 (66.7)	0.201	0.17	0.01	2.98
DLP n(%)		27 (90)	12 (92.3)	0.811	0.75	0.07	7.97
HTN n(%)		52(64.2)	12(52.2)	0.915	0.92	0.21	4.04
History DLP n(%)		26(40)	12(38.7)	0.904	1.06	0.44	2.54
History DM n(%)		24(36.9)	6(19.4)	0.082	2.44	0.88	6.79
History HTN n(%)		29(44.6)	13(41.9)	0.805	1.12	0.47	2.65

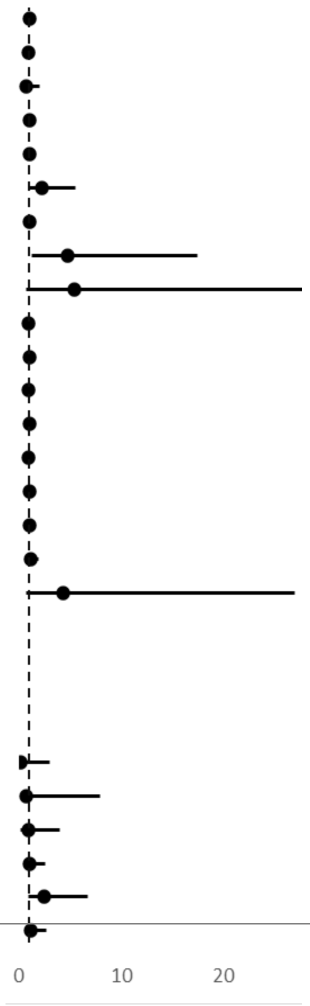

TableS5: Comparison of demographic variables and clinical characteristics between individuals with and without CAD in the Fars ethnic group

Variables	With CAD	Without CAD	P-Value	OR	25%	97.5%
Age	54.43±6.84	52.42±7.94	<0.001	1.04	1.02	1.05
BMI	28.07±5.1	28.5±5.3	0.11	0.99	0.97	1.00
WC	576 (56.3)	413 (63)	0.01	0.76	0.62	0.93
SBP	124.8±18.7	121.8±16.6	0.00	1.01	1.00	1.01
DBP	79.7±11.7	79.09±10.3	0.27	1.00	1.00	1.01
Sex (female) n(%)	303 (29.5)	415 (63.1)	<0.001	4.08	3.32	5.02
Sex (male) n(%)	724 (70.5)	243 (36.9)				
Economic	6.9±2.2	6.8±2	0.41	1.02	0.97	1.07
Smoke n(%)	458 (44.7)	148 (22.5)	<0.001	2.78	2.23	3.47
Opium n(%)	289 (28.2)	89 (13.6)	<0.001	2.50	1.93	3.25
Stress SSS	8.9±3.8	8.9±3.9	0.99	1.00	0.97	1.03
PE	7.9±3.1	7.8±3.1	0.88	1.00	0.97	1.04
PG	5.9±1.7	5.7±1.8	0.04	1.06	1.00	1.12
Acceptance	2.9±1.07	2.9±1.09	0.94	1.00	0.92	1.10
Avoidance	3.2±1.7	3.05±1.7	0.04	1.06	1.00	1.13
Anxiety.	8.2±4.7	10.05±4.7	<0.001	0.92	0.90	0.94
Depression	7.2±3.9	8.6±4.06	<0.001	0.91	0.89	0.94
Sex activity	6.3±1.9	5.8±1.8	<0.001	1.14	1.08	1.20
DM n(%)	356 (38.5)	149 (24.9)	<0.001	1.89	1.50	2.37
High FBS n(%)	230 (26.3)	82 (13.9)	<0.001	2.22	1.68	2.93
High LDL n(%)	87 (8.6)	107 (16.4)	<0.001	0.48	0.36	0.65
High Chol n(%)	55 (5.4)	48 (7.3)	0.12	0.73	0.49	1.08
High TG n(%)	201 (19.9)	110 (16.8)	0.11	1.23	0.95	1.59
Low HDL n(%)	571 (56.3)	327 (49.9)	0.01	1.29	1.06	1.57
DLP n(%)	810 (79.6)	505 (38.4)	0.22	1.16	0.92	1.47
HTN n(%)	476 (46.4)	279 (42.5)	0.11	1.17	0.96	1.43
History DLP n(%)	426 (41.5)	257 (39.1)	0.34	1.10	0.90	1.35
History DM n(%)	296 (28.8)	135 (20.5)	<0.001	1.57	1.25	1.98
History HTN n(%)	357 (34.8)	218 (33.1)	0.49	1.08	0.87	1.32

Odds ratio and 95% confidence interval

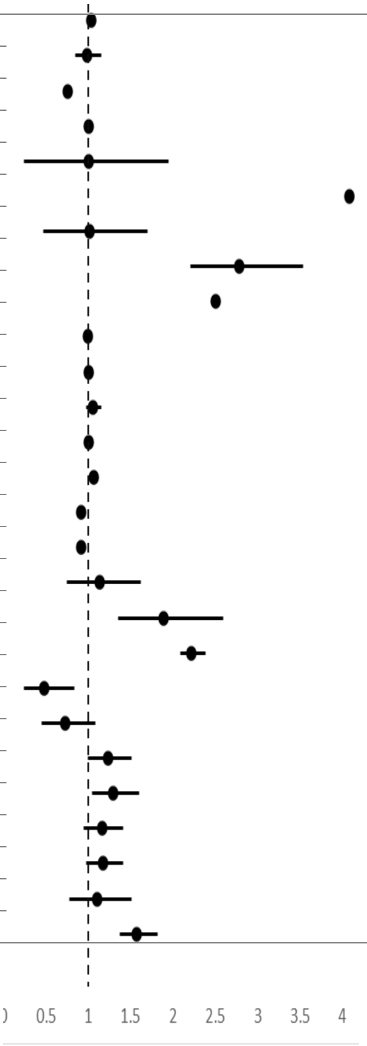

TableS6: Comparison of demographic variables and clinical characteristics between individuals with and without CAD in the Kurd ethnic group

Variables		With CAD	Without CAD	P-Value	OR	25%	97.5%
					Odds ratio and 95% confidence interval		
Age		55.20±6.76	51.43±8.61	<0.001	1.07	1.04	1.10
BMI		28.3±4.9	29.2±5.8	0.101	0.97	0.93	1.01
WC		104 (53.6)	112 (64)	0.043	0.65	0.43	0.99
SBP		112.1±16	114.3±16.1	0.194	0.99	0.98	1.00
DBP		70.1±11.6	71.8±10.7	0.158	0.99	0.97	1.01
Sex (female) n(%)		90 (46.4)	120 (68.2)	<0.001	2.48	1.62	3.79
Sex (male) n(%)		104 (53.6)	56 (31.8)				
Economic		6±2.3	5.8±1.98	0.348	1.05	0.95	1.15
Smoke n(%)		72(37.1)	37(21)	0.001	2.22	1.39	3.53
Opium n(%)		48(24.7)	26(14.9)	0.018	1.88	1.11	3.20
Stress	SSS	10±3.9	9.5±4.3	0.227	1.03	0.98	1.08
	PE	7.9±3	8±2.9	0.726	0.99	0.92	1.06
	PG	6.2±2.1	6.1±2.1	0.935	1.00	0.91	1.11
	Acceptance	2.8±1.3	2.9±1.2	0.403	0.93	0.79	1.10
	Avoidance	2.9±1.7	3.2±1.6	0.067	0.89	0.78	1.01
Anxiety.		9.1±4.7	10.5±4.4	0.004	0.94	0.90	0.98
Depression		7.6±3.8	8.9±3.8	0.001	0.91	0.86	0.96
Sex activity		6.4±2	5.9±2.01	0.016	1.14	1.02	1.26
DM n(%)		58 (30.4)	33 (19)	0.012	1.86	1.14	3.04
High FBS n(%)		43(22.5)	23(13.1)	0.02	1.92	1.10	3.34
High LDL n(%)		16 (8.3)	28 (16.1)	0.022	0.47	0.25	0.90
High Chol n(%)		8 (4.1)	9 (5.2)	0.64	0.79	0.30	2.10
High TG n(%)		53 (27.6)	39 (22.4)	0.253	1.32	0.82	2.13
Low HDL n(%)		115 (59.9)	99 (56.9)	0.561	1.13	0.75	1.72
DLP n(%)		153 (79.3)	126 (72.4)	0.124	1.46	0.90	2.36
HTN n(%)		83 (42.8)	66 (37.5)	0.301	1.25	0.82	1.89
History DLP n(%)		82(42.3)	55(31.4)	0.031	1.60	1.04	2.45
History DM n(%)		47(24.2)	25(14.3)	0.016	1.92	1.12	3.28
History HTN n(%)		78(40.2)	59(33.7)	0.197	1.32	0.86	2.02

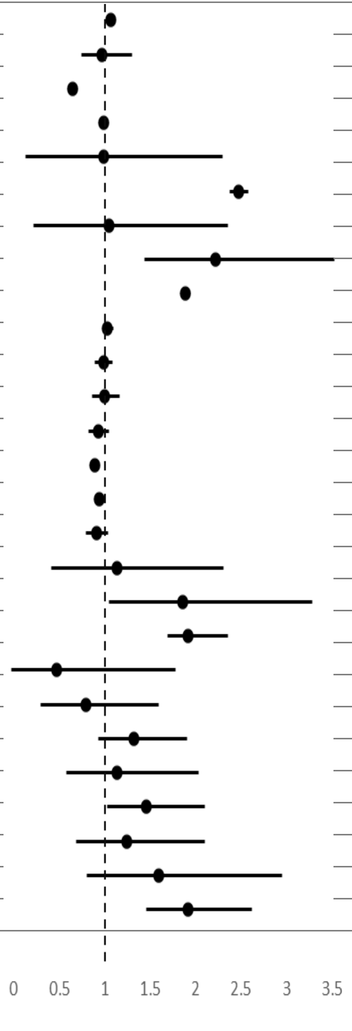

TableS7: Comparison of demographic variables and clinical characteristics between individuals with and without CAD in the Gilak ethnic group

Variables		With CAD	Without CAD	P-Value	Odds ratio and 95% confidence interval OR	25%	97.5%
Age		56.47±6.96	52.91±8.96	0.001	1.06	1.02	1.10
BMI		27.9±5.5	29.9±6.7	0.018	0.95	0.90	0.99
WC		77 (45.6)	49 (74.2)	<0.001	0.29	0.15	0.54
SBP		113.5±13.4	120.3±14.4	0.001	0.97	0.95	0.99
DBP		71.7±8.8	75.9±8.6	0.001	0.95	0.92	0.98
Sex (female) n(%)		87 (50.3)	43 (65.2)	0.039	1.85	1.03	3.33
Sex (male) n(%)		86(49.7)	23(34.8)				
Economic		4.5±2.2	4.8±1.9	0.362	0.94	0.82	1.08
Smoke n(%)		51(29.5)	12(18.2)	0.076	1.88	0.93	3.81
Opium n(%)		26(15)	5(7.6)	0.125	2.16	0.79	5.88
Stress	SSS	9.2±3	10±2.5	0.05	0.91	0.82	1.00
	PE	7.6±2.8	7.9±2.3	0.29	0.94	0.85	1.05
	PG	5.2±1.5	5.4±1.5	0.42	0.92	0.77	1.12
	Acceptance	2.4±0.95	2.4±0.7	0.96	1.01	0.73	1.39
	Avoidance	3.05±1.8	2.8±1.4	0.351	1.07	0.91	1.26
Anxiety.		6.7±4	7.3±3.4	0.302	0.96	0.89	1.04
Depression		7.3±2.7	8.01±2.8	0.091	0.91	0.82	1.01
Sex activity		6.3±1.4	5.9±1.03	**0.017**	1.26	1.01	1.58
DM n(%)		82 (48)	21 (32.3)	**0.03**	1.93	1.06	3.52
High FBS n(%)		67(39.2)	8(12.3)	**<0.001**	4.59	2.06	10.23
High LDL n(%)		22 (13.1)	8 (12.5)	0.904	1.05	0.44	2.51
High Chol n(%)		1 (4.1)	0 (0)	0.098			
High TG n(%)		46 (27.5)	8 (12.5)	**0.016**	2.66	1.18	6.01
Low HDL n(%)		99 (58.6)	39 (60.9)	0.744	0.91	0.50	1.63
DLP n(%)		137(80.1)	57 (87.7)	0.174	0.57	0.25	1.30
HTN n(%)		69(39.9)	38(57.6)	**0.014**	0.49	0.28	0.87
History DLP n(%)		81(46.8)	35(53)	0.39	0.78	0.44	1.38
History DM n(%)		61(35.3)	20(30.3)	0.469	1.25	0.68	2.31
History HTN n(%)		66(38.2)	36(54.5)	**0.022**	0.51	0.29	0.91

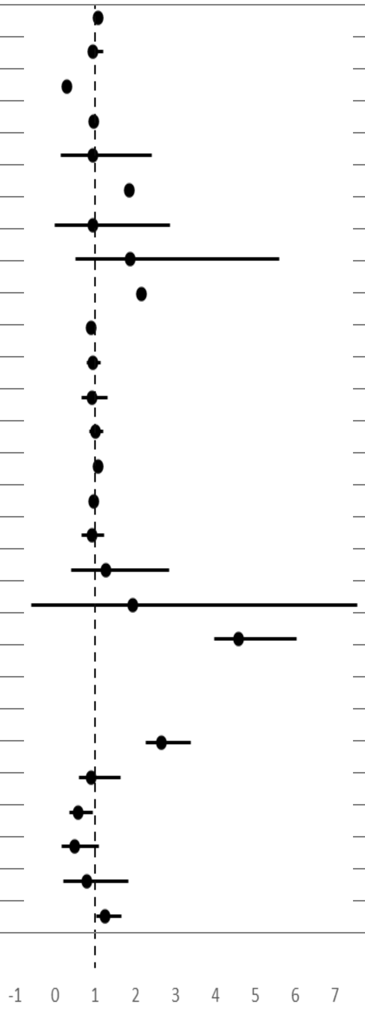

TableS8: Comparison of demographic variables and clinical characteristics between individuals with and without CAD in the Lur ethnic group

Variables		With CAD	Without CAD	P-Value	Odds ratio and 95% confidence interval		
					OR	25%	97.5%
Age		56.03±9.44	49.04±12.33	0.004	1.06	1.02	1.11
BMI		28.2±6.6	30.9±4.7	0.069	0.94	0.88	1.01
WC		51 (58)	18 (78.3)	0.074	0.38	0.13	1.12
SBP		133.7±17	126.9±22.9	0.125	1.02	0.99	1.05
DBP		82.5±8.3	82.2±11	0.891	1.00	0.95	1.06
Sex (female) n(%)		35 (39.8)	31 (56.5)	0.149	1.97	0.78	4.98
Sex (male) n(%)		53 (60.2)	10 (43.5)				
Economic		5.2±2.7	5.4±2	0.891	0.97	0.81	1.16
Smoke n(%)		36(40.9)	10(43.5)	0.824	0.90	0.36	2.28
Opium n(%)		27(30.7)	2(9.1)	0.04	4.43	0.97	20.29
Stress	SSS	9.6±3.2	8.6±4.9	0.262	1.07	0.95	1.21
	PE	8±2.8	6.5±3.4	0.037	1.18	1.01	1.38
	PG	5.4±1.8	5.3±2	0.922	1.01	0.79	1.30
	Acceptance	2.5±1.2	2.9±1.3	0.245	0.78	0.52	1.18
	Avoidance	3.2±1.8	3.7±1.9	0.227	0.86	0.67	1.10
Anxiety.		9.7±5.1	11.5±4.2	0.131	0.93	0.84	1.02
Depression		8.1±4.1	8.9±3.3	0.368	0.95	0.85	1.06
Sex activity		6.4±2.3	5.3±2.05	0.044	1.24	1.00	1.53
DM n(%)		28 (33.7)	5 (23.8)	0.383	1.63	0.54	4.91
High FBS n(%)		19(23.5)	1(5.3)	0.074	5.52	0.69	44.08
High LDL n(%)		4 (4.8)	5 (21.7)	0.009	0.18	0.04	0.74
High Chol n(%)		4 (4.8)	5 (21.7)	0.009	0.18	0.04	0.74
High TG n(%)		23 (27.4)	4 (17.4)	0.328	1.79	0.55	5.83
Low HDL n(%)		47 (56)	13 (56.5)	0.961	0.98	0.39	2.48
DLP n(%)		65 (77.4)	19 (82.6)	0.589	0.72	0.22	2.38
HTN n(%)		31(51.4)	23(41.1)	0.296	1.64	0.64	4.19
History DLP n(%)		32(36.4)	4(17.4)	0.084	2.71	0.85	8.68
History DM n(%)		20(22.7)	4(17.4)	0.58	1.40	0.43	4.58
History HTN n(%)		37(42)	7(30.4)	0.311	1.66	0.62	4.44

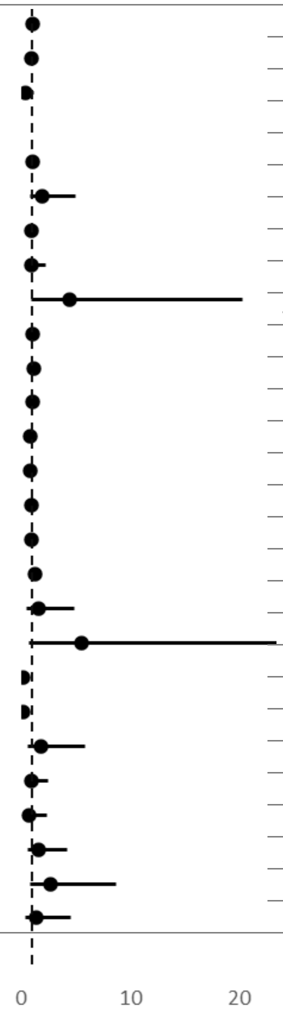

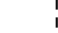

Supplement: Supplementary file 1 — contains Table S1-S8. [file jcvtr-17-49-s001.pdf]
